# Supplementary material for: The MR-Base platform supports systematic causal inference across the human phenome
Source: eLife. 2018 May 30;7:e34408. doi: 10.7554/eLife.34408 (PMC5976434; doi:10.7554/eLife.34408)
Supplement: Supplementary file 1. — (A) Genome-wide association studies with complete summary data in MR-Base as of December 2017. (B) List of Mendelian randomization analysis methods. (C) Genetic instruments for low density lipoprotein cholesterol. (D) Phenome-wide association study of LDL-C raising cardio-protective variant. (E) Limitations of Mendelian randomization and potential solutions. (F) Glossary of terms. (G) The schema of the MR-Base database. [file elife-34408-supp1.docx]

## **Supplementary tables**

### **Supplementary file 1A**. Genome-wide association studies with complete* summary data in MR-Base as of December 2017†

| **Consortium/ first author** | **Phenotype(s)** | **Pubmed ID** | **No. of GWAS analyses / traits** | **Max^+^ cases** | **Max^+^ controls / max^+^ sample size** | **Category** | **Subcategory** |
| --- | --- | --- | --- | --- | --- | --- | --- |
| ADIPOGen[^1^](https://paperpile.com/c/qJmYM5/yi4qP) | Adiponectin | 22479202 | 1 | NA | 39883 | Risk factor | Protein |
| Albagha[^2^](https://paperpile.com/c/qJmYM5/hpe0D) | Paget's disease | 21623375 | 1 | 741 | 3440 | Disease | Bone |
| AMDGene[^3^](https://paperpile.com/c/qJmYM5/CPduC) | Age-related macular degeneration | 23455636 | 3 | 7650 | 59494 | Disease | Eye |
| Baranzini[^4^](https://paperpile.com/c/qJmYM5/qel0b) | Multiple sclerosis | 19010793 | 1 | 978 | 1861 | Disease | Autoimmune / inflammatory |
| BioBank Japan Project[^5^](https://paperpile.com/c/qJmYM5/QnF0H) | C-reactive protein | 21196492 | 1 | NA | 10112 | Risk factor | Immune system |
| C4D[^6^](https://paperpile.com/c/qJmYM5/ef9vx) | Coronary heart disease | 21378988 | 1 | 15420 | 30482 | Disease | Cardiovascular |
| Capasso[^7^](https://paperpile.com/c/qJmYM5/Mp1LE) | Neuroblastoma | 23222812 | 1 | 1627 | 4881 | Disease | Cancer |
| CARDIoGRAM[^8^](https://paperpile.com/c/qJmYM5/3nqdT) | Coronary heart disease | 21378990 | 1 | 22233 | 86995 | Disease | Cardiovascular |
| CARDIoGRAMplusC4D[^9,10^](https://paperpile.com/c/qJmYM5/eeUr1+0rlYb) | Coronary heart disease | 26343387, 23202125 | 3 | 63746 | 194427 | Disease | Cardiovascular |
| Cha[^11^](https://paperpile.com/c/qJmYM5/brr2a) | Gallbladder cancer | 22318345 | 1 | 41 | 907 | Disease | Cancer |
| Chan[^12^](https://paperpile.com/c/qJmYM5/Si9EY) | Sitting height ratio | 25865494 | 4 | NA | 21590 | Risk factor | Anthropometric |
| Ciampa[^13^](https://paperpile.com/c/qJmYM5/xveDV) | Prostate cancer | 21372204 | 1 | 1175 | 2275 | Disease | Cancer |
| CKDGen[^14–17^](https://paperpile.com/c/qJmYM5/xhtBi+oFCo0+XeoJd+uZrJ8) | Kidney disease / markers of kidney function | 20383146, 26831199, 21355061, 26631737 | 15 | 12385 | 133814 | Disease | Kidney |
| Cordell[^18^](https://paperpile.com/c/qJmYM5/fRXNP) | Primary biliary cirrhosis | 26394269 | 1 | 2764 | 13239 | Disease | Autoimmune / inflammatory |
| CORNET[^19^](https://paperpile.com/c/qJmYM5/5FQh9) | Plasma cortisol | 25010111 | 1 | NA | 12597 | Risk factor | Hormone |
| DCCT & EDIC[^20^](https://paperpile.com/c/qJmYM5/CBRr8) | E-selectin | 19729612 | 1 | NA | 685 | Risk factor | Immune system |
| DIAGRAM[^21–23^](https://paperpile.com/c/qJmYM5/smrRD+etwmR+LoLks) | Type 2 diabetes | 26551672, 24509480, 22885922 | 4 | 27206 | 110452 | Disease | Diabetes |
| DIAGRAMplusMetabochip[^23^](https://paperpile.com/c/qJmYM5/LoLks) | Type 2 diabetes | 22885922 | 1 | 34840 | 149821 | Disease | Diabetes |
| Dubois[^24^](https://paperpile.com/c/qJmYM5/0nEtn) | Celiac disease | 20190752 | 2 | 4533 | 15283 | Disease | Autoimmune / inflammatory |
| Duerr[^25^](https://paperpile.com/c/qJmYM5/w6fkP) | Inflammatory bowel disease | 17068223 | 1 | 968 | 1963 | Disease | Autoimmune / inflammatory |
| EAGLE[^26–28^](https://paperpile.com/c/qJmYM5/e3zjk+XgdIr+z9FPo) | ADHD symptom scores, Aggression, Internalizing problems | 27663945, 26087016, 24839885 | 4 | NA | 18988 | Risk factor | Psychiatric / neurological |
| EAGLE Eczema[^29^](https://paperpile.com/c/qJmYM5/EBiES) | Eczema | 26482879 | 1 | 10788 | 40835 | Disease | Autoimmune / inflammatory |
| EGG[^30–34^](https://paperpile.com/c/qJmYM5/glVcd+hN1Em+q0n2C+wzEXk+MhI0s) | Birth anthropometrics | 23202124, 22504419, 22484627, 25281659, 23449627 | 13 | NA | 28459 | Risk factor | Anthropometric |
| ENIGMA[^35^](https://paperpile.com/c/qJmYM5/AX1Zq) | Subcortical brain structures | 25607358 | 8 | NA | 13193 | Risk factor | Psychiatric / neurological |
| Evans[^36^](https://paperpile.com/c/qJmYM5/CLvn4) | Copper, Selenium, Zinc | 23720494 | 8 | NA | 2874 | Risk factor | Trace elements |
| Feehally[^37^](https://paperpile.com/c/qJmYM5/kN0Sq) | IgA nephropathy | 20595679 | 1 | 977 | 5957 | Disease | Kidney |
| GABRIEL[^38^](https://paperpile.com/c/qJmYM5/l187G) | Asthma | 20860503 | 1 | 10365 | 26475 | Disease | Autoimmune / inflammatory |
| GCAN[^39^](https://paperpile.com/c/qJmYM5/emRM9) | Anorexia nervosa | 24514567 | 1 | 2907 | 17767 | Disease | Psychiatric / neurological |
| GEFOS[^40,41^](https://paperpile.com/c/qJmYM5/EIpdf+o7mAt) | Bone mineral density | 26367794, 22504420 | 5 | NA | 32961 | Risk factor | Bone |
| GIANT[^42–50^](https://paperpile.com/c/qJmYM5/6dGxl+adzmu+01y0R+4gbEd+BZqv3+ZCHMn+dDW7Y+GzG3z+M15tB) | Anthropometric traits | 25673413, 25673412, 20935630, 23563607, 20881960, 25282103, 23754948, 20935629, 22982992 | 71 | NA | 339224 | Risk factor | Anthropometric |
| GIS[^51^](https://paperpile.com/c/qJmYM5/k5AHQ) | Iron, Ferritin, Transferrin Saturation , Transferrin | 25352340 | 4 | NA | 23986 | Risk factor | Iron homeostasis |
| GLGC[^52^](https://paperpile.com/c/qJmYM5/p5Cz1) | Lipids | 24097068 | 8 | NA | 187365 | Risk factor | Lipid |
| GliomaScan[^53^](https://paperpile.com/c/qJmYM5/eNJmk) | Glioma | 22886559 | 2 | 1856 | 6811 | Disease | Cancer |
| Goris[^54^](https://paperpile.com/c/qJmYM5/qdtiu) | CSF antibody levels | 25616667 | 2 | NA | 3026 | Risk factor | Immune system |
| GPC[^55,56^](https://paperpile.com/c/qJmYM5/ck329+Pdm6n) | Personality | 21173776, 25993607 | 6 | NA | 160958 | Risk factor | Personality |
| GUGC[^57,58^](https://paperpile.com/c/qJmYM5/nrRBJ+z8I8r) | Gout, Urate | 23263486, 25811787 | 14 | 2115 | 110347 | Disease | Autoimmune / inflammatory |
| HaemGen[^59,60^](https://paperpile.com/c/qJmYM5/ACoFy+HwM03) | Haemotological traits | 22139419, 23222517 | 8 | NA | 71861 | Risk factor | Haemotological |
| Hofmann[^61^](https://paperpile.com/c/qJmYM5/35PRf) | Sarcoidosis | 22936702 | 1 | 637 | 1870 | Disease | Autoimmune / inflammatory |
| Hom[^62^](https://paperpile.com/c/qJmYM5/yCc9H) | Systemic lupus erythematosus | 18204098 | 1 | 1311 | 3094 | Disease | Autoimmune / inflammatory |
| Horikoshi[^63^](https://paperpile.com/c/qJmYM5/qRdw1) | Birth weight | 27680694 | 2 | NA | 153781 | Risk factor | Anthropometric |
| HRgene consortium[^64^](https://paperpile.com/c/qJmYM5/BhAAG) | Heart rate | 23583979 | 1 | NA | 92355 | Risk factor | Hemodynamic |
| IAC[^65^](https://paperpile.com/c/qJmYM5/SNxY7) | Abdominal aortic aneurysm | 27899403 | 1 | 4972 | 104830 | Disease | Cardiovascular |
| ICBP[^66,67^](https://paperpile.com/c/qJmYM5/P5bN+eiSwX) | Blood pressure | 21909115, 21909110 | 4 | NA | 74064 | Risk factor | Blood pressure |
| IGAP[^68^](https://paperpile.com/c/qJmYM5/U49uX) | Alzheimer's disease | 24162737 | 2 | 25580 | 74046 | Disease | Psychiatric / neurological |
| IIBDGC[^69–72^](https://paperpile.com/c/qJmYM5/XnSOT+ALE67+x1ytf+XV6rA) | Inflammatory bowel disease | 26192919, 21102463, 23128233, 21297633 | 20 | 31665 | 75000 | Disease | Autoimmune / inflammatory |
| ILCCO/TRICL[^73,74^](https://paperpile.com/c/qJmYM5/c10Iy+5vi1t) | Lung cancer | 24880342, 27488534 | 6 | 13479 | 56697 | Disease | Cancer |
| IMSGC[^75–77^](https://paperpile.com/c/qJmYM5/z0Upy+hS7Pb+g2IXh) | Multiple sclerosis | 17660530, 24076602, 21833088 | 3 | 14498 | 38589 | Disease | Autoimmune / inflammatory |
| ISGC[^78^](https://paperpile.com/c/qJmYM5/9bsLd) | Stroke | 26935894 | 4 | 10307 | 29633 | Disease | Cardiovascular |
| Jacobsen[^79^](https://paperpile.com/c/qJmYM5/UZRka) | Migraine in bipolar disorder | 25451450 | 1 | NA | 1374 | Risk factor | Psychiatric / neurological |
| Kettunen[^80^](https://paperpile.com/c/qJmYM5/0rHAe) | Metabolites | 27005778 | 123 | NA | 24925 | Metabolites | Multiple |
| Kiel[^81^](https://paperpile.com/c/qJmYM5/5DM7z) | Bone ultra sound attenuation | 17903296 | 1 | NA | 1141 | Risk factor | Bone |
| Kilpeläinen[^82^](https://paperpile.com/c/qJmYM5/zb3Aq) | Leptin | 26833098 | 2 | NA | 32161 | Risk factor | Hormone |
| Kohler[^83^](https://paperpile.com/c/qJmYM5/bZLQI) | Thyroid cancer | 23894154 | 1 | 649 | 1080 | Disease | Cancer |
| Li[^84^](https://paperpile.com/c/qJmYM5/0urR9) | Alzheimer's disease | 17998437 | 1 | 753 | 1489 | Disease | Psychiatric / neurological |
| Li[^85^](https://paperpile.com/c/qJmYM5/Mb0jv) | Upper gastrointestinal cancers | 23504502 | 1 | 3700 | 5811 | Disease | Cancer |
| Lu[^86^](https://paperpile.com/c/qJmYM5/dhV41) | Body fat | 26833246 | 1 | NA | 100716 | Risk factor | Anthropometric |
| Luciano[^87^](https://paperpile.com/c/qJmYM5/9SCdn) | Information processing speed | 21130836 | 8 | NA | 2956 | Risk factor | Information processing speed |
| MAGIC[^88–93^](https://paperpile.com/c/qJmYM5/AFrHI+fAku6+7QCnu+sTBMD+LzEAe+Djkdy) | Glycemic traits | 20081857, 20081858, 20858683, 24699409, 22581228, 22885924 | 24 | NA | 133010 | Risk factor | Glycemic |
| Maraganore[^94^](https://paperpile.com/c/qJmYM5/Ynygt) | Parkinson's disease | 16252231 | 1 | 443 | 886 | Disease | Psychiatric / neurological |
| Matarin[^95^](https://paperpile.com/c/qJmYM5/g1K0r) | Ischaemic stroke | 17434096 | 1 | 249 | 517 | Disease | Cardiovascular |
| MDACC[^96^](https://paperpile.com/c/qJmYM5/CSBz5) | Melanoma | 21926416 | 1 | 1804 | 2830 | Disease | Cancer |
| MESA[^97^](https://paperpile.com/c/qJmYM5/XJ63C) | Percent emphysema | 24383474 | 5 | NA | 7667 | Risk factor | Lung disease |
| Mueller[^98^](https://paperpile.com/c/qJmYM5/rBMw2) | Diabetic nephropathy | 16775037 | 1 | 1487 | 3079 | Disease | Kidney |
| Ober[^99^](https://paperpile.com/c/qJmYM5/ec1Ag) | Lp(a) Levels | 19124843 | 1 | NA | 357 | Risk factor | Lipid |
| Okada[^100^](https://paperpile.com/c/qJmYM5/YjYoC) | Rheumatoid arthritis | 24390342 | 2 | 14361 | 58284 | Disease | Autoimmune / inflammatory |
| Olfson[^101^](https://paperpile.com/c/qJmYM5/ZPTv7) | Alcohol dependence | 22978509 | 1 | NA | 3829 | Risk factor | Behavioural |
| Pankratz[^102^](https://paperpile.com/c/qJmYM5/twOrZ) | Parkinson's disease | 21829596 | 1 | 816 | 1672 | Disease | Psychiatric / neurological |
| PanScan1[^103^](https://paperpile.com/c/qJmYM5/aCrx8) | Pancreatic cancer | 19648918 | 1 | 1896 | 3835 | Disease | Cancer |
| PGC[^104–108^](https://paperpile.com/c/qJmYM5/oXs6c+AS7GJ+EQoAX+BgMV7+xH9cO) | Psychiatric diseases | 25056061, 20732625, 21926972, 23453885, 22472876 | 9 | 35476 | 82315 | Disease | Psychiatric / neurological |
| Project MinE[^109^](https://paperpile.com/c/qJmYM5/PE6qu) | Amyotrophic lateral sclerosis | 27455348 | 2 | 12577 | 36052 | Disease | Psychiatric / neurological |
| ReproGen[^110,111^](https://paperpile.com/c/qJmYM5/4LM9H+zeeda) | Reproductive aging | 25231870, 26414677 | 2 | NA | 182416 | Risk factor | Reproductive aging |
| Roederer[^112^](https://paperpile.com/c/qJmYM5/frvgW) | Immune cell subset frequency, Immune cell-surface protein expression levels | 25772697 | 151 | NA | 497 | Immune system | Immune cell subset frequency |
| Shin[^113^](https://paperpile.com/c/qJmYM5/TRbKI) | Metabolites | 24816252 | 452 | NA | 7822 | Metabolites | Multiple |
| Simon-Sanchez[^114^](https://paperpile.com/c/qJmYM5/4flA2) | Parkinson's disease | 19915575 | 1 | 1713 | 5691 | Disease | Psychiatric / neurological |
| Smith[^115^](https://paperpile.com/c/qJmYM5/9v7tv) | Bipolar disorder | 19488044 | 1 | 1346 | 3049 | Disease | Psychiatric / neurological |
| SSGAC[^116–120^](https://paperpile.com/c/qJmYM5/q1xUP+Qixan+WAADb+7A2pm+FNhWn) | Education, Reproductive behaviour | 23358156, 25201988, 23722424, 27089181, 27798627 | 17 | NA | 318863 | Risk factor | Education |
| Stahl[^121^](https://paperpile.com/c/qJmYM5/pno2B) | Rheumatoid arthritis | 20453842 | 1 | 5539 | 25708 | Disease | Autoimmune / inflammatory |
| TAG[^122^](https://paperpile.com/c/qJmYM5/tIeVE) | Smoking behaviours | 20418890 | 4 | NA | 74035 | Risk factor | Behavioural |
| Tang[^123^](https://paperpile.com/c/qJmYM5/txUT4) | Hirschsprung’s disease | 20361209 | 1 | 173 | 788 | Disease | Paediatric disease |
| Trynka[^124^](https://paperpile.com/c/qJmYM5/efXoj) | Celiac disease | 22057235 | 1 | 12041 | 24269 | Disease | Autoimmune / inflammatory |
| UK Biobank[^125–128^](https://paperpile.com/c/qJmYM5/Ved0a+QJ8rS+1VR5s+kG6Vy) | Multiple | 26961502, 27494321, 27015805, Neale lab | 605 | NA | 330000 | Multiple | Multiple |
| Wade[^129^](https://paperpile.com/c/qJmYM5/yAeqf) | Bulimia nervosa | 23568457 | 1 | 151 | 2442 | Disease | Psychiatric / neurological |

**Legend to Supplementary file 1A**. *complete refers to results for all single nucleotide polymorphisms reported in a GWAS analysis, with no exclusions on the basis of a p-value threshold for association with the target trait of interest. †Available studies are updated on a regular basis. ^+^Max refers to the maximum number of cases, maximum number of controls or maximum sample size available in a study. Study acronyms: **ADIPOGen** (Adiponectin genetics consortium), **AMD Gene** (Age-related Macular Degeneration Gene Consortium), **C4D** (Coronary Artery Disease Genetics Consortium), **CARDIoGRAM** (Coronary ARtery DIsease Genome wide Replication and Meta-analysis), **CKDGen** (Chronic Kidney Disease Genetics consortium), **CORNET** (The CORtisol NETwork), **DCCT/EDIC (**Diabetes Control and Complications Trial/Epidemiology of Diabetes Intervention and Complications study cohort), **DIAGRAM** (DIAbetes Genetics Replication And Meta-analysis), **EAGLE** (EArly Genetics & Lifecourse Epidemiology Eczema Consortium, excluding 23andMe), **EGG** (Early Growth Genetics Consortium), **ENIGMA** (Enhancing Neuro Imaging Genetics through Meta Analysis), **GABRIEL** (A Multidisciplinary Study to Identify the Genetic and Environmental Causes of Asthma in the European Community), **GCAN** (Genetic Consortium for Anorexia Nervosa), **GEFOS** (GEnetic Factors for OSteoporosis Consortium), **GIANT** (Genetic Investigation of ANthropometric Traits), **GIS** (Genetics of Iron Status), **GLGC** (Global Lipids Genetics Consortium), **GliomaScan** (cohort-based genome-wide association study of glioma), **GPC** (Genetics of Personality Consortium), **GUGC** (Global Urate and Gout consortium), **HaemGen** (haemotological and platelet traits genetics consortium), **HRgene** (heart rate genetics consortium), **IAC** (the International Aneurysm Consortium), **ICBP** (International Consortium for Blood Pressure), **IGAP** (International Genomics of Alzheimer's Project), **IIBDGC** (International Inflammatory Bowel Disease Genetics Consortium), **ILCCO/TRICL** (International Lung Cancer Consortium/Transdisciplinary Research in Cancer of the Lung), **IMSGC** (International Multiple Sclerosis Genetic Consortium), **ISGC** (International Stroke Genetics Consortium), **MAGIC** (Meta-Analyses of Glucose and Insulin-related traits Consortium), **MDACC** (MD Anderson Cancer Center), **MESA** (Multi-Ethnic Study of Atherosclerosis), **PanScan** (Pancreatic Cancer Cohort Consortium), **PGC** (Psychiatric Genomics Consortium), **ReproGen** (Reproductive Genetics Consortium), **SSGAC** (Social Science Genetics Association Consortium) and **TAG** (Tobacco and Genetics Consortium). **Other acronyms**: ADHD, attention deficit hyperactivity disorder; GWAS, genome-wide association study.

### **Supplementary file 1B.** List of Mendelian randomization analysis methods

| **Function** | **Citation** | **Description** |
| --- | --- | --- |
| *Methods to estimate exposure-outcome effects in TwoSampleMR package* | | |
| IVW  linear regression, using fixed or multiplicative random effects models | Johnson[^130^](https://paperpile.com/c/qJmYM5/wCf0Q); Ehret et al[^66^](https://paperpile.com/c/qJmYM5/P5bN); Burgess et al[^131^](https://paperpile.com/c/qJmYM5/BcFhP); Dastani et al[^1^](https://paperpile.com/c/qJmYM5/yi4qP); Burgess et al[^132^](https://paperpile.com/c/qJmYM5/VdjkT) | >1 SNP; effect of exposure on outcome estimated by linear regression of BYG on BXG, with SNPs weighted by 1/seBYG^2 and the intercept constrained to pass through zero. In the random effects IVW model, the standard error of the causal estimate (the slope from the regression model) Fixed and random effects IVW models have identical slopes but the variance of the random effects model is inflated by Q/(K-1) (if this quantity is greater than 1), where Q is Cochran’s heterogeneity statistic and K is the number of instrumental SNPs. The random effects model allows heterogeneity in causal estimates and horizontal pleiotropy if the horizontal pleiotropy is balanced around the instrumental SNPs, i.e. is not directional. |
| Maximum likelihood | Pierce and Burgess[^133^](https://paperpile.com/c/qJmYM5/tRX4K) | >1 SNP; uses maximum likelihood to fit a linear model between BXG and BYG that best fits the data. Similar to the fixed effects IVW approach, the method assumes that the effect of the exposure on the outcome due to each SNP is the same, i.e. assumes there is no heterogeneity or horizontal pleiotropy. An unbiased estimate will be returned in the absence of horizontal pleiotropy or when horizontal pleiotropy is balanced (but the variance of the effect estimate will be overly precise). An advantage of the method is that it may provide more reliable results in the presence of measurement error in the SNP-exposure effect. |
| Median estimator, weighted or unweighted | Bowden et al[^134^](https://paperpile.com/c/qJmYM5/Z8M2m) | >2 SNPs; assumes that the IV assumptions hold for genetic variants making up at least 50% of the information in the analysis (in an unweighted analysis this corresponds to half the instruments); weights derived from the inverse of the standard error of the Wald ratio estimated by the delta method[^135^](https://paperpile.com/c/qJmYM5/B0Vyd). |
| Mode-based estimators, weighted or unweighted | Hartwig et al[^136^](https://paperpile.com/c/qJmYM5/bxYAi) | >3 SNPs; clusters the instruments into groups based on similarity of causal effects, and selects the causal effect estimate from the cluster that has the largest number  of instruments. Returns a valid causal effect if the instruments in the largest cluster are valid.  Standard errors are obtained by bootstrapping. Can be implemented with or without the assumption of no measurement error in the exposure estimates (NOME).  The simple mode is the unweighted mode of the empirical density function of causal estimates, whereas the weighted mode is weighted by the inverse variance of the outcome effect. Requires the user to selected a bandwidth parameter (set to 1 by default). |
| Multivariable Mendelian randomization | Burgess et al[^137,138^](https://paperpile.com/c/qJmYM5/kRpRM+uJznt) | >3 SNPs; models effect of multiple exposures on a single outcome, where the exposures share some of the same instruments. |
| MR-Egger regression | Bowden et al[^139^](https://paperpile.com/c/qJmYM5/Juf6J) | >2 SNPs; same as IVW linear regression except that intercept is not constrained to pass through zero; standard errors can be obtained either directly from the regression model or by parametric bootstrap. Allows heterogeneity in causal estimates and directional horizontal pleiotropy (but horizontal pleiotropy effects must not correlate with the SNP-exposure effects, also known as the InSIDE assumption). |
| Penalized weighted median function | Bowden et al[^134^](https://paperpile.com/c/qJmYM5/Z8M2m) | >2 SNPs; Weighted median approach but downweights variants with substantially heterogeneous variant-specific causal estimates. |
| Rucker framework | Bowden et al[^140^](https://paperpile.com/c/qJmYM5/l9U2w) | >3 SNPs. This is a framework for navigating between different models of pleiotropy to obtain less biased effect sizes and appropriate standard errors. A fixed effects IVW model assumes no horizontal pleiotropy, no bias due to the omission of the intercept term and no heterogeneity. Evidence of heterogeneity would suggest a random effects model is more appropriate, and if heterogeneity can be reduced by moving from IVW to Egger then the Egger model is accepted. If heterogeneity is still present in the Egger model then it is implemented in a random effects framework. MR-Base uses a multiplicative random effects model. |
| Wald ratio | Lawlor et al[^141^](https://paperpile.com/c/qJmYM5/qFWgz) | Single SNP available; BYG/BXG; standard error = seBYG/BXG |
| *Methods to estimate exposure-outcome effects in other R packages* | | |
| Interface with MendelianRandomization R package | Yavorska and Burgess[^142^](https://paperpile.com/c/qJmYM5/iYzNI) | This package implements a non-overlapping set of methods with the TwoSampleMR package, methods that accounts for linkage disequilibrium between instruments. |
| MR-PRESSO (Mendelian Randomization Pleiotropy RESidual Sum and Outlier) | Verbanck et al [^143^](https://paperpile.com/c/qJmYM5/YVKiu) | A method for the detection and correction for outliers in results based on the IVW method. |
| mr.raps (Mendelian randomization using the robust adjusted profile score) | Zhao et al[^144^](https://paperpile.com/c/qJmYM5/Nu7BG) | A method that can use many weak instruments and correct for horizontal pleiotropy based on robust adjusted profile scores |
| *Other statistical tests in the TwoSampleMR package* | | |
| Heterogeneity between causal effect estimates | Greco et al[^145^](https://paperpile.com/c/qJmYM5/1ba7A); Pierce and Burgess[^133^](https://paperpile.com/c/qJmYM5/tRX4K) | Tests for heterogeneity using Cochran’s Q (for meta-analysis, IVW and MR-Egger methods) or likelihood ratio test (for maximum likelihood method) |
| Intercept test from MR-Egger regression | Bowden et al[^139^](https://paperpile.com/c/qJmYM5/Juf6J) | The intercept from MR-Egger regression can be interpreted as an estimate of the average pleiotropic effect across instruments. An intercept term that differs from zero is indicative of overall directional pleiotropy. Tests the null hypothesis that the intercept is zero. |
| *Diagnostic plots in the TwoSampleMR package* | | |
| Forest plots | - | Visualise the contribution of each SNP to the overall estimate. Plots the effect of the exposure on the outcome using each SNP as a separate instrument or combined into a single instrument; can be used to visualise heterogeneity between causal effect estimates (e.g. due to horizontal pleiotropy) |
| Funnel plots | Sterne et al[^146^](https://paperpile.com/c/qJmYM5/t2qGG); Bowden et al[^139^](https://paperpile.com/c/qJmYM5/Juf6J) | Plots instrument strength against the causal effect (estimated using each SNP as a separate instrument). Symmetry indicates that the strongest instrument provides an estimate that is closest to the average estimated effect, with weaker instruments spread evenly on both sides. Asymmetry is indicative of invalid instruments (e.g. due to horizontal pleiotropy) |
| Leave-one-out plots | - | Plot the results of leave-one-out analysis, where each SNP is sequentially excluded. Used to identify sensitivity of the analysis to a single SNP driving the result |
| Scatter plots | - | Plot the SNP-exposure effect (BXG) against the SNP-outcome (BYG effect). Visualise outliers and agreement of causal estimates used by different estimation methods. Deviation from a linear dose-response relationship, or with an intercept that does not pass through zero, could be indicative of violations of IV assumptions). The intercept from MR-Egger regression[^139^](https://paperpile.com/c/qJmYM5/Juf6J) tests the null hypothesis that the intercept is zero. |
| *Diagnostic plots in the RadialMR package* | | |
| Radial plots | Bowden et al[^147^](https://paperpile.com/c/qJmYM5/qbXrQ) | Fit and visualise the results of radial inverse variance weighted and radial MR-Egger models. Can be used to detect outliers and influential data points |

**Legend to Supplementary file 1B.** BXG, effect of the SNP on the exposure; BYG, effect of the SNP on the outcome; se, standard error, SNP, single nucleotide polymorphism; MR, Mendelian randomization; IV, instrumental variable assumption; IVW, inverse-variance weighted. The assumptions are: (IV1) the instrument is associated with the exposure; (IV2) the instrument is independent of the outcome conditional on the exposure and confounders (also known as the ‘exclusion restriction’ assumption); and (IV3) the instrument is independent of known and unknown confounders. See the glossary of terms at the end of the supplement for an explanation of MR concepts. Supplementary table 5 summarises MR limitations.

###

###

### **Supplementary file 1C**. Genetic instruments for low density lipoprotein cholesterol

| **SNP** | **Gene** | **Chr** | **Position** | **Effect allele** | **Other allele** | **EAF** | **beta** | **se** | **pval** |
| --- | --- | --- | --- | --- | --- | --- | --- | --- | --- |
| rs12027135 | LDLRAP1 | 1 | 25775732 | A | T | 0.46 | -0.03 | 0.004 | 2.00E-14 |
| rs12748152 | PIGV | 1 | 27138392 | T | C | 0.09 | 0.05 | 0.007 | 3.00E-12 |
| rs2479409 | PCSK9 | 1 | 55504649 | G | A | 0.32 | 0.064 | 0.004 | 3.00E-50 |
| rs2131925 | ANGPTL3 | 1 | 63025941 | G | T | 0.34 | -0.049 | 0.004 | 3.00E-32 |
| rs629301 | SORT1 | 1 | 109818305 | G | T | 0.24 | -0.167 | 0.005 | 1.00E-200 |
| rs267733 | ANXA9 | 1 | 150958835 | G | A | 0.16 | -0.033 | 0.006 | 5.00E-09 |
| rs2642442 | MOSC1 | 1 | 220973562 | C | T | 0.33 | -0.036 | 0.005 | 5.00E-11 |
| rs514230 | IRF2BP2 | 1 | 234858596 | A | T | 0.48 | -0.036 | 0.005 | 9.00E-12 |
| rs1367117 | APOB | 2 | 21263899 | A | G | 0.32 | 0.119 | 0.004 | 1.00E-182 |
| rs4299376 | ABCG5 | 2 | 44072575 | G | T | 0.31 | 0.081 | 0.005 | 4.00E-72 |
| rs2710642 | EHBP1 | 2 | 63149556 | G | A | 0.35 | -0.024 | 0.004 | 6.00E-09 |
| rs10490626 | INSIG2 | 2 | 118835840 | A | G | 0.08 | -0.051 | 0.007 | 2.00E-12 |
| rs2030746 | LOC84931 | 2 | 121309487 | T | C | 0.4 | 0.021 | 0.004 | 9.00E-09 |
| rs1250229 | FN1 | 2 | 216304383 | T | C | 0.27 | -0.024 | 0.004 | 3.00E-08 |
| rs11563251 | UGT1A1 | 2 | 234679383 | T | C | 0.12 | 0.034 | 0.006 | 5.00E-08 |
| rs7640978 | CMTM6 | 3 | 32533009 | T | C | 0.09 | -0.039 | 0.007 | 1.00E-08 |
| rs17404153 | ACAD11 | 3 | 132163199 | T | G | 0.14 | -0.034 | 0.006 | 2.00E-09 |
| rs6831256 | LRPAP1 | 4 | 3473138 | G | A | 0.42 | 0.022 | 0.004 | 2.00E-08 |
| rs12916 | HMGCR | 5 | 74656538 | C | T | 0.4 | 0.073 | 0.004 | 8.00E-78 |
| rs4530754 | CSNK1G3 | 5 | 122855415 | G | A | 0.46 | -0.028 | 0.004 | 4.00E-12 |
| rs6882076 | TIMD4 | 5 | 156390296 | T | C | 0.36 | -0.046 | 0.004 | 3.00E-31 |
| rs3177928 | HLA | 6 | 3673308 | A | G | 0.17 | 0.045 | 0.005 | 3.00E-17 |
| rs3177928 | HLA | 6 | 3690553 | A | G | 0.17 | 0.045 | 0.005 | 3.00E-17 |
| rs3177928 | HLA | 6 | 3754501 | A | G | 0.17 | 0.045 | 0.005 | 3.00E-17 |
| rs3177928 | HLA | 6 | 3759104 | A | G | 0.17 | 0.045 | 0.005 | 3.00E-17 |
| rs3177928 | HLA | 6 | 3791214 | A | G | 0.17 | 0.045 | 0.005 | 3.00E-17 |
| rs3177928 | HLA | 6 | 3882852 | A | G | 0.17 | 0.045 | 0.005 | 3.00E-17 |
| rs3757354 | MYLIP | 6 | 16127406 | T | C | 0.24 | -0.038 | 0.004 | 2.00E-17 |
| rs1800562 | HFE | 6 | 26093140 | A | G | 0.07 | -0.062 | 0.008 | 8.00E-14 |
| rs3177928 | HLA | 6 | 32412434 | A | G | 0.17 | 0.045 | 0.005 | 3.00E-17 |
| rs9488822 | FRK | 6 | 116312892 | T | A | 0.36 | 0.031 | 0.006 | 2.00E-07 |
| rs1564348 | LPA | 6 | 160578859 | C | T | 0.18 | 0.048 | 0.005 | 3.00E-21 |
| rs12670798 | DNAH11 | 7 | 21607351 | C | T | 0.25 | 0.034 | 0.005 | 5.00E-14 |
| rs4722551 | MIR148A | 7 | 25991825 | C | T | 0.2 | 0.039 | 0.005 | 4.00E-14 |
| rs2072183 | NPC1L1 | 7 | 44579179 | C | G | 0.29 | 0.039 | 0.005 | 7.00E-16 |
| rs9987289 | PPP1R3B | 8 | 9183357 | A | G | 0.1 | -0.071 | 0.007 | 9.00E-24 |
| rs10102164 | SOX17 | 8 | 55421613 | A | G | 0.21 | 0.032 | 0.005 | 4.00E-11 |
| rs2081687 | CYP7A1 | 8 | 59388564 | T | C | 0.36 | 0.031 | 0.006 | 1.00E-07 |
| rs2954029 | TRIB1 | 8 | 126490971 | T | A | 0.47 | -0.056 | 0.004 | 2.00E-50 |
| rs11136341 | PLEC1 | 8 | 145043542 | G | A | 0.4 | 0.045 | 0.007 | 7.00E-12 |
| rs3780181 | VLDLR | 9 | 2640758 | G | A | 0.08 | -0.044 | 0.007 | 2.00E-09 |
| rs2255141 | GPAM | 10 | 113933885 | A | G | 0.3 | 0.03 | 0.004 | 1.00E-13 |
| rs174546 | FADS1 | 11 | 61569829 | T | C | 0.36 | -0.051 | 0.004 | 2.00E-39 |
| rs964184 | APOA1 | 11 | 116648916 | C | G | 0.84 | -0.086 | 0.008 | 2.00E-26 |
| rs11220462 | ST3GAL4 | 11 | 126243951 | A | G | 0.14 | 0.059 | 0.006 | 7.00E-21 |
| rs11065987 | BRAP | 12 | 112072423 | G | A | 0.41 | -0.027 | 0.004 | 1.00E-11 |
| rs1169288 | HNF1A | 12 | 121416649 | C | A | 0.34 | 0.038 | 0.004 | 6.00E-21 |
| rs4942486 | BRCA2 | 13 | 32953387 | T | C | 0.48 | 0.024 | 0.004 | 2.00E-11 |
| rs8017377 | NYNRIN | 14 | 24883886 | A | G | 0.46 | 0.03 | 0.004 | 3.00E-15 |
| rs3764261 | CETP | 16 | 56993323 | A | C | 0.32 | -0.053 | 0.004 | 2.00E-34 |
| rs2000999 | HPR | 16 | 72108092 | A | G | 0.2 | 0.065 | 0.005 | 4.00E-41 |
| rs314253 | DLG4 | 17 | 7091649 | C | T | 0.37 | -0.024 | 0.004 | 3.00E-10 |
| rs7206971 | OSBPL7 | 17 | 45425114 | A | NA | 0.49 | 0.029 | 0.006 | 3.00E-07 |
| rs1801689 | APOH | 17 | 64210579 | C | NA | 0.04 | 0.103 | 0.015 | 1.00E-11 |
| rs6511720 | LDLR | 19 | 11202305 | T | G | 0.12 | -0.221 | 0.006 | 1.00E-200 |
| rs10401969 | CILP2 | 19 | 19407717 | C | T | 0.09 | -0.118 | 0.008 | 3.00E-54 |
| rs4420638 | APOE | 19 | 45422945 | G | A | 0.19 | 0.225 | 0.008 | 2.00E-178 |
| rs364585 | SPTLC3 | 20 | 12962717 | A | G | 0.38 | -0.025 | 0.004 | 4.00E-10 |
| rs2328223 | SNX5 | 20 | 17845920 | C | NA | 0.21 | 0.03 | 0.005 | 6.00E-09 |
| rs2902940 | MAFB | 20 | 39091486 | G | A | 0.3 | -0.027 | 0.004 | 2.00E-11 |
| rs6029526 | TOP1 | 20 | 39672617 | A | T | 0.47 | 0.044 | 0.005 | 5.00E-18 |
| rs5763662 | MTMR3 | 22 | 30378702 | T | C | 0.04 | 0.077 | 0.013 | 1.00E-08 |
| rs4253772 | PPARA | 22 | 46627602 | T | C | 0.11 | 0.031 | 0.006 | 3.00E-08 |

**Legend to Supplementary file 1C.** Betas correspond to the standard deviation change in low density lipoprotein (LDL) cholesterol per copy of the effect allele. EAF, effect allele frequency; SNP, single nucleotide polymorphism; se, standard error; Chr, chromosome; position, base pair position

### **Supplementary file 1D.** Phenome-wide association study of LDL-C raising cardio-protective variant

|  | SNP-trait effect^†^ | | | Trait-CHD effect^‡^ | |
| --- | --- | --- | --- | --- | --- |
| Trait | beta (SE) | P value | PMID | beta (SE) | P value |
| Coronary heart disease*[^148^](https://paperpile.com/c/qJmYM5/mkdJ) | -0.06 (0.011) | 2.50E-08 | 26343387 | NA | NA |
| LDL cholesterol[^149^](https://paperpile.com/c/qJmYM5/jINV) | 0.027 (0.004) | 1.20E-11 | 24097068 | 0.011 (0.001) | 1.04E-13 |
| Body mass index[^150^](https://paperpile.com/c/qJmYM5/XwtI) | 0.015 (0.003) | 1.01E-06 | 25673413 | 0.007 (0.001) | 4.03E-14 |
| Hip circumference[^151^](https://paperpile.com/c/qJmYM5/DTtX) | 0.018 (0.004) | 6.80E-07 | 25673412 | 0.004 (0.002) | 2.35E-02 |
| Systolic blood pressure[^66^](https://paperpile.com/c/qJmYM5/P5bN) | NA | 3.43E-12 | 21909115 | NA | 1.31E-10 |
| Diastolic blood pressure[^66^](https://paperpile.com/c/qJmYM5/P5bN) | NA | 2.12E-08 | 21909115 | NA | 6.44E-10 |
| Mean arterial pressure[^152^](https://paperpile.com/c/qJmYM5/Sv9i) | NA | 1.82E-09 | 21909110 | NA | 4.32E-05 |
| Red blood cell count[^153^](https://paperpile.com/c/qJmYM5/TwsJ) | -0.028 (0.005) | 4.21E-08 | 23222517 | -0.001 (0.003) | 7.25E-01 |
| Haemoglobin concentration[^153^](https://paperpile.com/c/qJmYM5/TwsJ) | -0.034 (0.005) | 1.73E-13 | 23222517 | 0.002 (0.005) | 6.62E-01 |
| Hematocrit[^154^](https://paperpile.com/c/qJmYM5/ynFq) | 0.051 (0.007) | 1.00E-12 | 19862010 | -0.012 (0.005) | 2.54E-02 |
| Packed cell volume[^153^](https://paperpile.com/c/qJmYM5/TwsJ) | -0.02 (0.003) | 1.51E-10 | 23222517 | 0.004 (0.008) | 6.22E-01 |
| Platelet count[^155^](https://paperpile.com/c/qJmYM5/6JNm) | -0.067 (0.007) | 4.94E-21 | 22139419 | -0.003 (0.003) | 4.19E-01 |
| C-glycosyltryptophan^¶^[^156^](https://paperpile.com/c/qJmYM5/y8Dd) | -0.081 (0.019) | 1.71E-05 | 24816252 | -0.001 (0.003) | 8.51E-01 |
| Erythronate[^156^](https://paperpile.com/c/qJmYM5/y8Dd) | -0.08 (0.016) | 8.30E-07 | 24816252 | -0.002 (0.004^+^) | 6.27E-01 |
| Kynurenine[^156^](https://paperpile.com/c/qJmYM5/y8Dd) | -0.119 (0.015) | 2.36E-14 | 24816252 | -0.011 (0.011) | 3.12E-01 |
| Serum cystatin C (eGFRcys) | 0.043 (0.008) | 6.60E-07 | 26831199 | -0.004 (0.004) | 3.33E-01 |
| Urate[^157^](https://paperpile.com/c/qJmYM5/6XBn) | -0.025 (0.004) | 2.58E-08 | 23263486 | -0.003 (0.001) | 6.21E-02 |
| Inflammatory bowel disease*[^158^](https://paperpile.com/c/qJmYM5/08dL) | -0.048 (0.01) | 1.71E-06 | 26192919 | 0 (0.001) | 9.97E-01 |
| Crohn's disease*[^158^](https://paperpile.com/c/qJmYM5/08dL) | -0.059 (0.012) | 1.14E-06 | 26192919 | 0 (0.001) | 5.60E-01 |
| Rheumatoid arthritis*[^159^](https://paperpile.com/c/qJmYM5/TDF7) | -0.073 (0.016) | 4.90E-06 | 24390342 | -0.001 (0.001) | 2.51E-01 |
| Primary biliary cirrhosis*[^160^](https://paperpile.com/c/qJmYM5/BHLJ) | -0.174 (0.031) | 3.00E-08 | 26394269 | -0.003 (0.001) | 9.13E-02 |
| Celiac disease*[^161^](https://paperpile.com/c/qJmYM5/WwKs) | -0.148 (0.029) | 2.56E-07 | 20190752 | -0.005 (0.005) | 3.37E-01 |
| Tetralogy of Fallot*[^162^](https://paperpile.com/c/qJmYM5/VRWk) | -0.296 (0.045) | 8.00E-11 | 23297363 | -0.004 (0.005^+^) | 3.93E-01 |

**Legend to Supplementary file 1D.** ^†^Effect of SNP on trait, where beta refers to standard deviation or log odds ratio change in the trait per copy of the LDL raising allele. *binary traits where SNP-trait effect is a log odds ratio. ^‡^Effect of selected trait on coronary heart disease estimated by inverse variance weighted (IVW) linear regression or Wald ratio (when only a single SNP was available to instrument the trait^¶^), where beta refers to log odds ratio for coronary heart disease per unit change in the trait, scaled to reflect the magnitude of the SNP-trait effect. Standard errors for the effect from IVW linear regression were estimated using a multiplicative random effects model, except where there was underdispersion in the causal estimates between SNPs, in which case they were estimated using a fixed effects model^+^. The selected traits were identified through a search of rs11065987, rs1250229 and rs4530754 in the MR-Base database of complete summary data and the MR-Base standardised version of the GWAS catalog, excluding associations with p value ≥2.04e-05 (0.05 / 2453 ‘trait lookups’). rs1250229 and rs4530754 were not associated with non-lipid non-vascular-disease traits at this threshold and were therefore excluded from further evaluation. LDL-C, low density lipoprotein cholesterol; NA, not available; SE, standard error; PMID, PubMed identifier.

### **Supplementary file 1E**. Limitations of Mendelian randomization and potential solutions

This limitations below are provided for quick reference only and we encourage readers to consult the cited references for further details. Overviews of Mendelian randomization limitations can also be found in existing reviews[^141,163–169^](https://paperpile.com/c/qJmYM5/pUgaU+yVAMT+Pu1aF+XcciT+EXlFZ+iZBCI+mxkr4+qFWgz)

| **Limitation** | **Potential Impact** | **Solution** |
| --- | --- | --- |
| Binary exposures[^170–172^](https://paperpile.com/c/qJmYM5/040ro+iRHnX+PcppA)  For example, user wishes to instrument disease status using SNPs that associate with log odds of disease | Causal interpretation unclear because the instrument does not reflect presence or absence of the trait but liability or propensity to develop the trait. Associations may therefore reflect a shared genetic architecture or shared determinants of disease risk. | Users should acknowledge limitation and avoid making strong causal interpretations. |
| Canalization[^164,165^](https://paperpile.com/c/qJmYM5/yVAMT+Pu1aF) (when developmental processes buffer the phenotypic impact of a genetic variant) | Causal hypothesis true but due to developmental buffer a valid instrument is not associated with the outcome. | Using instruments derived from different stages of the life-course may help. |
| Collider bias[^169,173,174^](https://paperpile.com/c/qJmYM5/OUM4p+mxkr4+DMNQv) (when adjustment for, or stratification on, a trait that is the causal consequence of the exposure and outcome induces an erroneous association between the exposure and outcome) | If analysis is adjusted for a collider this may induce an erroneous association between exposure and outcome. Stratification (e.g. analyses run separately for obese or non-obese individuals) or selection bias (e.g. study participants more likely to be healthy) can also induce collider bias. | Difficult to avoid when using summary data (as done in MR-Base), as 2SMR analyses will inherit all adjustments made in the original GWAS. Users should assess the scope for collider bias by assessing which variables were adjusted for, or stratified on, in the original GWAS. Further advice on how to deal with collider bias can be found in Roher[^174^](https://paperpile.com/c/qJmYM5/DMNQv), Paternoster et al[^169^](https://paperpile.com/c/qJmYM5/mxkr4) and Munafò et al[^173^](https://paperpile.com/c/qJmYM5/OUM4p). |
| Correlation with confounders[^164,165^](https://paperpile.com/c/qJmYM5/yVAMT+Pu1aF) | The instrument is associated with confounders of the exposure-outcome association. Violation of IV assumption 3. | Assess the association of the instrument with potential known confounders (but cannot rule out associations with unknown confounders). |
| Data dredging (researchers run all possible analyses and prioritise those results with the smallest p-values for publication) | Increases the likelihood of false positive findings and publication bias. | Researchers should adhere to analysis plans and be transparent about which analyses they run, including exploratory analyses. |
| Dynastic effects[^175^](https://paperpile.com/c/qJmYM5/8JcHy), when the exposure trait in a previous generation influences the environment of the current generation | Introduces confounding into a Mendelian randomization study. | Alternative study designs, such as studies of sibling pairs, can control for confounding due to dynastic effects[^175^](https://paperpile.com/c/qJmYM5/8JcHy). |
| Exposure heterogeneity[^164^](https://paperpile.com/c/qJmYM5/yVAMT), where there may be multiple components or intercorrelated features to an exposure, only some of which are instrumentable  For example, lipoproteins are strongly intercorrelated and can vary substantially in size, molar concentration and cholesterol and triglyceride content[^176,177^](https://paperpile.com/c/qJmYM5/BSQhK+uFJ6b). Smoking behaviour varies in cigarettes smoked per day, tobacco exposure, depth of inhalation, etc[^178,179^](https://paperpile.com/c/qJmYM5/eCK7P+iBBqi). | Makes it difficult to infer which aspect or component of an exposure or group of strongly correlated exposures is driving causality with the outcome. May bias the magnitude of the estimated effect. | When present, causal interpretations should be relaxed, for example restricted to inferences about directions of effect or that the exposure pathway (e.g. HDL-related pathways but not HDL cholesterol per se[^137^](https://paperpile.com/c/qJmYM5/kRpRM)) is causally relevant.  Multivariable Mendelian randomization can be used to adjust for intercorrelations amongst closely related exposures. |
| False positive instrument (instrument does not affect the target exposure, violation of IV assumption 1) | If causal hypothesis is true, biases exposure-outcome effect towards the null. | Define instrument using genotype-phenotype associations that have been replicated by independent studies. |
| Frailty effects[^180^](https://paperpile.com/c/qJmYM5/BkGAt) | Induces associations for late-onset traits | Simulation studies can be used to assess the likely magnitude of the bias[^181,182^](https://paperpile.com/c/qJmYM5/jnVxe+yhjzR) |
| Horizontal pleiotropy[^164,165,167^](https://paperpile.com/c/qJmYM5/yVAMT+EXlFZ+Pu1aF) (instrument is associated with multiple traits via separate pathways, potentially introducing an alternative pathway to the outcome that does not include the hypothesized exposure, violation of IV assumption 2) | Incorrect inference of a causal effect of the exposure on the outcome | Several tools exist to evaluate the sensitivity of causal estimates to this problem, for example: MR-Egger regression, Rucker framework, weighted-median estimator, mode-based estimator, MR-PRESSO, MR-RAPS, heterogeneity statistics, funnel plots, leave-one-out analyses and radial plots (see Supplementary table 2 for further details). |
| Instrument based on a single or small number of genetic variants | Severely constrains ability to run sensitivity analyses to appraise IV assumptions. | Users should avoid making causal claims when only a single genetic variant is available. Causal inference may be possible if a plausible biological mechanism is known (e.g. using genetic variants at the C-reactive protein [CRP] gene as proxies for CRP). |
| Instrument-exposure effect differs between blood and sites of disease[^164^](https://paperpile.com/c/qJmYM5/yVAMT)  For example, it is known that genetic variants may affect biomarkers in opposite directions in blood and disease sites[^183–185^](https://paperpile.com/c/qJmYM5/8ahnO+zzvLf+5Y56M) but biomarker measurements are typically blood-based. | Incorrect inferences about magnitudes and directions of effect | Potentially avoidable through assessment of eQTLs in blood and disease-relevant tissues, (e.g. using GTEx[^186^](https://paperpile.com/c/qJmYM5/0omA4) available via the MRInstruments package in MR-Base), or through detailed biological understanding of the genetic pathways involved |
| Low variation between instrument-exposure effects, or large standard errors in the instrument-exposure effect estimates[^187^](https://paperpile.com/c/qJmYM5/0h4LT) | MR Egger estimates will be biased towards the null due to regression dilution bias | MR Egger I^2^ statistic can be calculated to evaluate the magnitude of dilution bias. For example, an I^2^ of 90% will lead to a 10% underestimation of the effect size. SIMEX (simulation extrapolation) correction can be used to account for regression dilution bias.[^187^](https://paperpile.com/c/qJmYM5/0h4LT) |
| Non-linear association between exposure and outcome[^188^](https://paperpile.com/c/qJmYM5/n71kI) | Inferences about the magnitude of exposure-outcome effects typically require that the shape of the association between exposure and outcome is linear. If the causal relationship is non-linear, then causal effects should be interpreted as reflecting the population average causal effect and may not be generalisable to exposure subgroups, such as the extreme ends of the exposure distribution. | Methods available to estimate non-linear causal effects but these require individual level data and cannot be performed in MR-Base.[^188,189^](https://paperpile.com/c/qJmYM5/ZJvtB+n71kI) |
| Power[^164,190^](https://paperpile.com/c/qJmYM5/Cm1ZZ+yVAMT) (analyses may be subject to low statistical power, due to the low phenotypic variance typically explained by genetic variants, if the causal effect is small, or there is substantial measurement error in the outcome) | Could lead to false negative findings. | Confidence intervals around the effect estimate can be used to assess the uncertainty in the estimate. Using multiple genetic variants to define an instrument could increase the variance explained in the exposure and, therefore, power. Researchers should run power calculations prior to conducting analyses. |
| Reverse causal instruments[^191^](https://paperpile.com/c/qJmYM5/qHP9x), where the effect of genetic variants on a hypothesized exposure actually occurs via the hypothesized outcome | Leads to incorrect inferences about directions of causality. | 1) Bi-directional Mendelian randomization can be used, where effect is assessed in both directions[^192^](https://paperpile.com/c/qJmYM5/zqgtT). An effect in only one direction helps rule out the presence of reverse causal instruments.  2) The Steiger test can be used to estimate the direction of causation. This is implemented in the TwoSampleMR package in the mr_steiger function.[^193^](https://paperpile.com/c/qJmYM5/g0VYt) |
|  |  |  |
| Trait transformations that limit generalisability of results to applied settings  Phenotypes are often subjected to transformations, e.g. log, Z or inverse rank normal transformations, prior to GWAS analyses. The summary data from such analyses will therefore reflect the effect of the SNP on the phenotype on the transformed scale. | Limits the ability to extrapolate from Mendelian randomization findings to applied settings, e.g. in predicting the impact of an intervention on disease incidence in a clinical trial.  For example, results based on log transformed exposure data will reflect changes on a multiplicative or doubling exposure scale, which could correspond to large absolute changes that are difficult to achieve with existing drugs in a clinical trial. | Estimate the instrument-exposure effect on the original untransformed scale. Requires access to individual level data or summary data derived from analyses of untransformed data. |
| Exposure and outcome studies are not from the same population[^164^](https://paperpile.com/c/qJmYM5/yVAMT) | May bias the magnitude of the causal effect estimate (but may still be possible to infer the direction of an effect). | The samples used to define the instrument-exposure and instrument-outcome associations in a two-sample Mendelian randomization analysis should be drawn from similar populations, practically defined as being of similar age and sex distribution and ancestral/geographic region of origin. |
| Exposure and outcome studies contain overlapping participants[^133,164^](https://paperpile.com/c/qJmYM5/tRX4K+yVAMT) | May bias causal effect towards the confounded observational association (an example of weak instrument bias) | Strong instruments (in practice defined as an F statistic >10 for the instrument-exposure association) are less susceptible to this bias. Users should quantify the degree of overlap between their exposure and outcome studies[^133^](https://paperpile.com/c/qJmYM5/tRX4K). |
| Weak instruments bias[^133^](https://paperpile.com/c/qJmYM5/tRX4K), where effect of the instrument on the exposure is estimated in a small sample and therefore subject to uncertainty and chance correlations with confounders. | If the exposure and outcome studies overlap, weak instruments may bias causal effect estimates towards the confounded observational association. If samples are independent, uncertainty in the instrument-exposure effects may bias causal effect estimates towards the null. | Assess the scope of the problem by estimating sample overlap between studies as well as as F statistics for the instrument-exposure association[^133^](https://paperpile.com/c/qJmYM5/tRX4K). Not usually an issue in practice because instruments tend to be based on ‘GWAS hits’, defined using stringent significance thresholds (e.g. P<5e-8), although Winner’s curse bias may lead to overestimation of instrument-exposure effects. |
| Winner’s curse[^164,194^](https://paperpile.com/c/qJmYM5/iHQID+yVAMT), where instrument-exposure effect estimates are overestimated in the discovery study | Winner’s curse compounds the effect of weak instruments bias, biasing causal effect estimates towards the confounded observational association in the presence of sample overlap between the discovery and outcome studies. If studies are independent, Winner’s curse can bias causal effect estimates towards the null. | Use replication studies to define the instrument. However, if the replication study is much smaller than the discovery GWAS, SNP-exposure effects will be estimated with less precision, which could re-introduce weak instruments bias. |

**Legend to Supplementary file 1E.** GWAS, genome-wide association study; IV, instrumental variable; 2SMR, 2-sample Mendelian; MR-PRESSO (MR Pleiotropy RESidual Sum and Outlier), MR-RAPS (MR using the robust adjusted profile score)

### Supplementary file 1F. Glossary of terms

| Collider bias | When statistical adjustment for a trait (*the collider*) that is a downstream consequence of both the exposure X and the outcome Y induces an association between X and Y that was not previously present[^174^](https://paperpile.com/c/qJmYM5/DMNQv). If published genetic associations with the exposure and/or outcome are adjusted for a collider, this may lead to collider bias in the MR analysis. Stratification of analyses can also induce collider bias (e.g. running models separately for obese or non-obese individuals is equivalent to adjusting for obesity). |
| --- | --- |
| Confounding | When the relationship between an exposure and outcome is not causal but is due to the effects of a third variable (the confounder) on the exposure and the outcome. |
| Exposure | Any trait that is the cause of another trait. In MR, this can be very broadly defined. May include molecular traits like gene expression, DNA-methylation, metabolites and proteins, as well as more complex traits, including cholesterol, body mass index, smoking and education. |
| Instrument or instrumental variable | A genetic variant, typically a SNP, used as a proxy for the exposure in a MR study. A valid instrument is associated with the exposure (IV assumption 1), is associated with the outcome exclusively via its effect on the exposure (IV assumption 2) and is not associated with confounders of the exposure-outcome association (IV assumption 3). See Figure 1b for a visualisation of the IV assumptions. |
| Mendelian randomization | A technique to estimate the effect of an exposure on an outcome using genetic variants as instruments or proxies for the exposure |
| Outcome | The trait that is the hypothesized consequence of the exposure. Outcomes can be very broadly defined. May include molecular traits like gene expression, DNA-methylation, metabolites and proteins, as well as more complex traits, including cholesterol, body mass index, smoking, education and disease. |
| Pleiotropy | Occurs when a genetic variant is associated with multiple traits. Vertical pleiotropy occurs when the traits are all on the same pathway (a requirement for MR studies). Horizontal pleiotropy, when multiple traits are associated with the genetic variant via separate pathways, can introduce violations of IV assumption 2. |
| Summary data | Results from a genetic association study, including the beta coefficient, standard error and p values from a regression model, and metrics of study quality (e.g. tests for Hardy-Weinberg equilibrium, Cochran’s Q test for heterogeneity and imputation info or r^2^ scores). Also includes information on modelled effect alleles and effect allele frequency. |
| Weak instrument bias | Bias due to uncertainty in SNP-exposure association that correlates with uncertainty in the SNP-outcome association (due to sample overlap). Biases causal effect estimates towards the confounded observational association. The bias is compounded by small samples and Winner’s curse. Instruments strongly correlated with the exposure (in practice defined as F statistic >10) are less susceptible to the bias. |
| Winner’s curse | In discovery GWAS, the phenomenon of overestimating genetic effect sizes for ‘lead’ SNPs, when these are selected on the basis of the smallest P value[^164^](https://paperpile.com/c/qJmYM5/yVAMT). |

**Legend to Supplementary file 1F.** GWAS, genome-wide association study; IV, instrumental variable; SNP, single nucleotide polymorphism; MR, Mendelian randomization

## Supplementary figures

### Supplementary file 1G. The schema of the MR-Base database


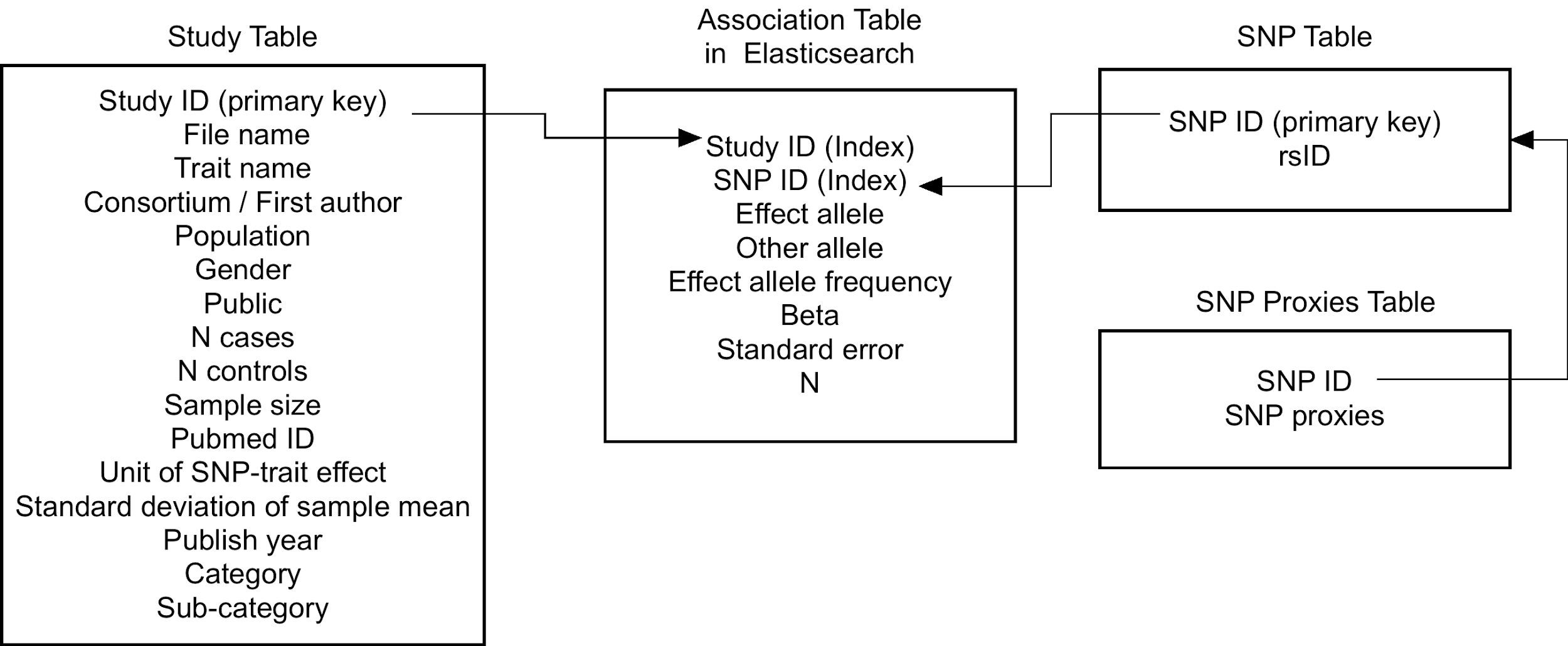


**Legend to Supplementary file 1G.** The black line with arrow means the Study ID and SNP ID are the foreign keys (links) to structure these four tables. Category refers to manually curated trait category (e.g. whether disease or risk factor); access level refers to whether the data is publically available or is restricted (e.g. to members of a consortium); beta (effect estimate for the SNP from a linear or logistic regression model); N, sample size; SNP, single nucleotide polymorphism; effect allele refers to the modelled or coded allele in an additive regression model in a GWAS; other allele refers to the non-effect allele;

## References

1. Dastani, Z. *et al.* Novel loci for adiponectin levels and their influence on type 2 diabetes and metabolic traits: a multi-ethnic meta-analysis of 45,891 individuals. *PLoS Genet.* **8,** e1002607 (2012).

2. Albagha, O. M. E. *et al.* Genome-wide association identifies three new susceptibility loci for Paget’s disease of bone. *Nat. Genet.* **43,** 685–689 (2011).

3. Fritsche, L. G. *et al.* Seven new loci associated with age-related macular degeneration. *Nat. Genet.* **45,** 433–9, 439e1–2 (2013).

4. Baranzini, S. E. *et al.* Genome-wide association analysis of susceptibility and clinical phenotype in multiple sclerosis. *Hum. Mol. Genet.* **18,** 767–778 (2009).

5. Okada, Y. *et al.* Genome-wide association study for C-reactive protein levels identified pleiotropic associations in the IL6 locus. *Hum. Mol. Genet.* **20,** 1224–1231 (2011).

6. Coronary Artery Disease (C4D) Genetics Consortium. A genome-wide association study in Europeans and South Asians identifies five new loci for coronary artery disease. *Nat. Genet.* **43,** 339–344 (2011).

7. Capasso, M. *et al.* Replication of GWAS-identified neuroblastoma risk loci strengthens the role of BARD1 and affirms the cumulative effect of genetic variations on disease susceptibility. *Carcinogenesis* **34,** 605–611 (2013).

8. Schunkert, H. *et al.* Large-scale association analysis identifies 13 new susceptibility loci for coronary artery disease. *Nat. Genet.* **43,** 333–338 (2011).

9. Nikpay, M. *et al.* A comprehensive 1,000 Genomes-based genome-wide association meta-analysis of coronary artery disease. *Nat. Genet.* **47,** 1121–1130 (2015).

10. CARDIoGRAMplusC4D Consortium *et al.* Large-scale association analysis identifies new risk loci for coronary artery disease. *Nat. Genet.* **45,** 25–33 (2013).

11. Cha, P.-C. *et al.* A genome-wide association study identifies SNP in DCC is associated with gallbladder cancer in the Japanese population. *J. Hum. Genet.* **57,** 235–237 (2012).

12. Chan, Y. *et al.* Genome-wide Analysis of Body Proportion Classifies Height-Associated Variants by Mechanism of Action and Implicates Genes Important for Skeletal Development. *Am. J. Hum. Genet.* **96,** 695–708 (2015).

13. Ciampa, J. *et al.* Large-scale exploration of gene-gene interactions in prostate cancer using a multistage genome-wide association study. *Cancer Res.* **71,** 3287–3295 (2011).

14. Köttgen, A. *et al.* New loci associated with kidney function and chronic kidney disease. *Nat. Genet.* **42,** 376–384 (2010).

15. Pattaro, C. *et al.* Genetic associations at 53 loci highlight cell types and biological pathways relevant for kidney function. *Nat. Commun.* **7,** 10023 (2016).

16. Böger, C. A. *et al.* CUBN is a gene locus for albuminuria. *J. Am. Soc. Nephrol.* **22,** 555–570 (2011).

17. Teumer, A. *et al.* Genome-wide Association Studies Identify Genetic Loci Associated With Albuminuria in Diabetes. *Diabetes* **65,** 803–817 (2016).

18. Cordell, H. J. *et al.* International genome-wide meta-analysis identifies new primary biliary cirrhosis risk loci and targetable pathogenic pathways. *Nat. Commun.* **6,** 8019 (2015).

19. Bolton, J. L. *et al.* Genome wide association identifies common variants at the SERPINA6/SERPINA1 locus influencing plasma cortisol and corticosteroid binding globulin. *PLoS Genet.* **10,** e1004474 (2014).

20. Paterson, A. D. *et al.* Genome-wide association identifies the ABO blood group as a major locus associated with serum levels of soluble E-selectin. *Arterioscler. Thromb. Vasc. Biol.* **29,** 1958–1967 (2009).

21. Gaulton, K. J. *et al.* Genetic fine mapping and genomic annotation defines causal mechanisms at type 2 diabetes susceptibility loci. *Nat. Genet.* **47,** 1415–1425 (2015).

22. DIAbetes Genetics Replication And Meta-analysis (DIAGRAM) Consortium *et al.* Genome-wide trans-ancestry meta-analysis provides insight into the genetic architecture of type 2 diabetes susceptibility. *Nat. Genet.* **46,** 234–244 (2014).

23. Morris, A. P. *et al.* Large-scale association analysis provides insights into the genetic architecture and pathophysiology of type 2 diabetes. *Nat. Genet.* **44,** 981–990 (2012).

24. Dubois, P. C. A. *et al.* Multiple common variants for celiac disease influencing immune gene expression. *Nat. Genet.* **42,** 295–302 (2010).

25. Duerr, R. H. *et al.* A genome-wide association study identifies IL23R as an inflammatory bowel disease gene. *Science* **314,** 1461–1463 (2006).

26. Middeldorp, C. M. *et al.* A Genome-Wide Association Meta-Analysis of Attention-Deficit/Hyperactivity Disorder Symptoms in Population-Based Pediatric Cohorts. *J. Am. Acad. Child Adolesc. Psychiatry* **55,** 896–905.e6 (2016).

27. Pappa, I. *et al.* A genome-wide approach to children’s aggressive behavior: The EAGLE consortium. *Am. J. Med. Genet. B Neuropsychiatr. Genet.* **171,** 562–572 (2016).

28. Benke, K. S. *et al.* A genome-wide association meta-analysis of preschool internalizing problems. *J. Am. Acad. Child Adolesc. Psychiatry* **53,** 667–676.e7 (2014).

29. Paternoster, L. *et al.* Multi-ancestry genome-wide association study of 21,000 cases and 95,000 controls identifies new risk loci for atopic dermatitis. *Nat. Genet.* **47,** 1449–1456 (2015).

30. Horikoshi, M. *et al.* New loci associated with birth weight identify genetic links between intrauterine growth and adult height and metabolism. *Nat. Genet.* **45,** 76–82 (2013).

31. Taal, H. R. *et al.* Common variants at 12q15 and 12q24 are associated with infant head circumference. *Nat. Genet.* **44,** 532–538 (2012).

32. Bradfield, J. P. *et al.* A genome-wide association meta-analysis identifies new childhood obesity loci. *Nat. Genet.* **44,** 526–531 (2012).

33. van der Valk, R. J. P. *et al.* A novel common variant in DCST2 is associated with length in early life and height in adulthood. *Hum. Mol. Genet.* **24,** 1155–1168 (2015).

34. Cousminer, D. L. *et al.* Genome-wide association and longitudinal analyses reveal genetic loci linking pubertal height growth, pubertal timing and childhood adiposity. *Hum. Mol. Genet.* **22,** 2735–2747 (2013).

35. Hibar, D. P. *et al.* Common genetic variants influence human subcortical brain structures. *Nature* **520,** 224–229 (2015).

36. Evans, D. M. *et al.* Genome-wide association study identifies loci affecting blood copper, selenium and zinc. *Hum. Mol. Genet.* **22,** 3998–4006 (2013).

37. Feehally, J. *et al.* HLA has strongest association with IgA nephropathy in genome-wide analysis. *J. Am. Soc. Nephrol.* **21,** 1791–1797 (2010).

38. Moffatt, M. F. *et al.* A large-scale, consortium-based genomewide association study of asthma. *N. Engl. J. Med.* **363,** 1211–1221 (2010).

39. Boraska, V. *et al.* A genome-wide association study of anorexia nervosa. *Mol. Psychiatry* **19,** 1085–1094 (2014).

40. Zheng, H.-F. *et al.* Whole-genome sequencing identifies EN1 as a determinant of bone density and fracture. *Nature* **526,** 112–117 (2015).

41. Estrada, K. *et al.* Genome-wide meta-analysis identifies 56 bone mineral density loci and reveals 14 loci associated with risk of fracture. *Nat. Genet.* **44,** 491–501 (2012).

42. Locke, A. E. *et al.* Genetic studies of body mass index yield new insights for obesity biology. *Nature* **518,** 197–206 (2015).

43. Shungin, D. *et al.* New genetic loci link adipose and insulin biology to body fat distribution. *Nature* **518,** 187–196 (2015).

44. Speliotes, E. K. *et al.* Association analyses of 249,796 individuals reveal 18 new loci associated with body mass index. *Nat. Genet.* **42,** 937–948 (2010).

45. Berndt, S. I. *et al.* Genome-wide meta-analysis identifies 11 new loci for anthropometric traits and provides insights into genetic architecture. *Nat. Genet.* **45,** 501–512 (2013).

46. Lango Allen, H. *et al.* Hundreds of variants clustered in genomic loci and biological pathways affect human height. *Nature* **467,** 832–838 (2010).

47. Wood, A. R. *et al.* Defining the role of common variation in the genomic and biological architecture of adult human height. *Nat. Genet.* **46,** 1173–1186 (2014).

48. Randall, J. C. *et al.* Sex-stratified genome-wide association studies including 270,000 individuals show sexual dimorphism in genetic loci for anthropometric traits. *PLoS Genet.* **9,** e1003500 (2013).

49. Heid, I. M. *et al.* Meta-analysis identifies 13 new loci associated with waist-hip ratio and reveals sexual dimorphism in the genetic basis of fat distribution. *Nat. Genet.* **42,** 949–960 (2010).

50. Yang, J. *et al.* FTO genotype is associated with phenotypic variability of body mass index. *Nature* **490,** 267–272 (2012).

51. Benyamin, B. *et al.* Novel loci affecting iron homeostasis and their effects in individuals at risk for hemochromatosis. *Nat. Commun.* **5,** 4926 (2014).

52. Global Lipids Genetics Consortium *et al.* Discovery and refinement of loci associated with lipid levels. *Nat. Genet.* **45,** 1274–1283 (2013).

53. Rajaraman, P. *et al.* Genome-wide association study of glioma and meta-analysis. *Hum. Genet.* **131,** 1877–1888 (2012).

54. Goris, A. *et al.* Genetic variants are major determinants of CSF antibody levels in multiple sclerosis. *Brain* **138,** 632–643 (2015).

55. de Moor, M. H. M. *et al.* Meta-analysis of genome-wide association studies for personality. *Mol. Psychiatry* **17,** 337–349 (2012).

56. Genetics of Personality Consortium *et al.* Meta-analysis of Genome-wide Association Studies for Neuroticism, and the Polygenic Association With Major Depressive Disorder. *JAMA Psychiatry* **72,** 642–650 (2015).

57. Köttgen, A. *et al.* Genome-wide association analyses identify 18 new loci associated with serum urate concentrations. *Nat. Genet.* **45,** 145–154 (2013).

58. Huffman, J. E. *et al.* Modulation of genetic associations with serum urate levels by body-mass-index in humans. *PLoS One* **10,** e0119752 (2015).

59. Gieger, C. *et al.* New gene functions in megakaryopoiesis and platelet formation. *Nature* **480,** 201–208 (2011).

60. van der Harst, P. *et al.* Seventy-five genetic loci influencing the human red blood cell. *Nature* **492,** 369–375 (2012).

61. Hofmann, S. *et al.* Genome-wide association analysis reveals 12q13.3-q14.1 as new risk locus for sarcoidosis. *Eur. Respir. J.* **41,** 888–900 (2013).

62. Hom, G. *et al.* Association of systemic lupus erythematosus with C8orf13-BLK and ITGAM-ITGAX. *N. Engl. J. Med.* **358,** 900–909 (2008).

63. Horikoshi, M. *et al.* Genome-wide associations for birth weight and correlations with adult disease. *Nature* **538,** 248–252 (2016).

64. den Hoed, M. *et al.* Identification of heart rate-associated loci and their effects on cardiac conduction and rhythm disorders. *Nat. Genet.* **45,** 621–631 (2013).

65. Jones, G. T. *et al.* Meta-Analysis of Genome-Wide Association Studies for Abdominal Aortic Aneurysm Identifies Four New Disease-Specific Risk Loci. *Circ. Res.* **120,** 341–353 (2017).

66. Ehret, G. B. *et al.* Genetic variants in novel pathways influence blood pressure and cardiovascular disease risk. *Nature* **478,** 103–109 (2011).

67. Wain, L. V. *et al.* Genome-wide association study identifies six new loci influencing pulse pressure and mean arterial pressure. *Nat. Genet.* **43,** 1005–1011 (2011).

68. Lambert, J. C. *et al.* Meta-analysis of 74,046 individuals identifies 11 new susceptibility loci for Alzheimer’s disease. *Nat. Genet.* **45,** 1452–1458 (2013).

69. Liu, J. Z. *et al.* Association analyses identify 38 susceptibility loci for inflammatory bowel disease and highlight shared genetic risk across populations. *Nat. Genet.* **47,** 979–986 (2015).

70. Franke, A. *et al.* Genome-wide meta-analysis increases to 71 the number of confirmed Crohn’s disease susceptibility loci. *Nat. Genet.* **42,** 1118–1125 (2010).

71. Jostins, L. *et al.* Host-microbe interactions have shaped the genetic architecture of inflammatory bowel disease. *Nature* **491,** 119–124 (2012).

72. Anderson, C. A. *et al.* Meta-analysis identifies 29 additional ulcerative colitis risk loci, increasing the number of confirmed associations to 47. *Nat. Genet.* **43,** 246–252 (2011).

73. Wang, Y. *et al.* Rare variants of large effect in BRCA2 and CHEK2 affect risk of lung cancer. *Nat. Genet.* **46,** 736–741 (2014).

74. Patel, Y. M. *et al.* Novel Association of Genetic Markers Affecting CYP2A6 Activity and Lung Cancer Risk. *Cancer Res.* **76,** 5768–5776 (2016).

75. International Multiple Sclerosis Genetics Consortium *et al.* Risk alleles for multiple sclerosis identified by a genomewide study. *N. Engl. J. Med.* **357,** 851–862 (2007).

76. International Multiple Sclerosis Genetics Consortium (IMSGC) *et al.* Analysis of immune-related loci identifies 48 new susceptibility variants for multiple sclerosis. *Nat. Genet.* **45,** 1353–1360 (2013).

77. International Multiple Sclerosis Genetics Consortium *et al.* Genetic risk and a primary role for cell-mediated immune mechanisms in multiple sclerosis. *Nature* **476,** 214–219 (2011).

78. Malik, R. *et al.* Low-frequency and common genetic variation in ischemic stroke: The METASTROKE collaboration. *Neurology* **86,** 1217–1226 (2016).

79. Jacobsen, K. K. *et al.* Genome wide association study identifies variants in NBEA associated with migraine in bipolar disorder. *J. Affect. Disord.* **172,** 453–461 (2015).

80. Kettunen, J. *et al.* Genome-wide study for circulating metabolites identifies 62 loci and reveals novel systemic effects of LPA. *Nat. Commun.* **7,** 11122 (2016).

81. Kiel, D. P. *et al.* Genome-wide association with bone mass and geometry in the Framingham Heart Study. *BMC Med. Genet.* **8 Suppl 1,** S14 (2007).

82. Kilpeläinen, T. O. *et al.* Genome-wide meta-analysis uncovers novel loci influencing circulating leptin levels. *Nat. Commun.* **7,** 10494 (2016).

83. Köhler, A. *et al.* Genome-wide association study on differentiated thyroid cancer. *J. Clin. Endocrinol. Metab.* **98,** E1674–81 (2013).

84. Li, H. *et al.* Candidate single-nucleotide polymorphisms from a genomewide association study of Alzheimer disease. *Arch. Neurol.* **65,** 45–53 (2008).

85. Li, W.-Q. *et al.* Genetic variants in DNA repair pathway genes and risk of esophageal squamous cell carcinoma and gastric adenocarcinoma in a Chinese population. *Carcinogenesis* **34,** 1536–1542 (2013).

86. Lu, Y. *et al.* New loci for body fat percentage reveal link between adiposity and cardiometabolic disease risk. *Nat. Commun.* **7,** 10495 (2016).

87. Luciano, M. *et al.* Whole genome association scan for genetic polymorphisms influencing information processing speed. *Biol. Psychol.* **86,** 193–202 (2011).

88. Saxena, R. *et al.* Genetic variation in GIPR influences the glucose and insulin responses to an oral glucose challenge. *Nat. Genet.* **42,** 142–148 (2010).

89. Dupuis, J. *et al.* New genetic loci implicated in fasting glucose homeostasis and their impact on type 2 diabetes risk. *Nat. Genet.* **42,** 105–116 (2010).

90. Soranzo, N. *et al.* Common variants at 10 genomic loci influence hemoglobin A₁(C) levels via glycemic and nonglycemic pathways. *Diabetes* **59,** 3229–3239 (2010).

91. Prokopenko, I. *et al.* A central role for GRB10 in regulation of islet function in man. *PLoS Genet.* **10,** e1004235 (2014).

92. Manning, A. K. *et al.* A genome-wide approach accounting for body mass index identifies genetic variants influencing fasting glycemic traits and insulin resistance. *Nat. Genet.* **44,** 659–669 (2012).

93. Scott, R. A. *et al.* Large-scale association analyses identify new loci influencing glycemic traits and provide insight into the underlying biological pathways. *Nat. Genet.* **44,** 991–1005 (2012).

94. Maraganore, D. M. *et al.* High-resolution whole-genome association study of Parkinson disease. *Am. J. Hum. Genet.* **77,** 685–693 (2005).

95. Matarín, M. *et al.* A genome-wide genotyping study in patients with ischaemic stroke: initial analysis and data release. *Lancet Neurol.* **6,** 414–420 (2007).

96. Amos, C. I. *et al.* Genome-wide association study identifies novel loci predisposing to cutaneous melanoma. *Hum. Mol. Genet.* **20,** 5012–5023 (2011).

97. Manichaikul, A. *et al.* Genome-wide study of percent emphysema on computed tomography in the general population. The Multi-Ethnic Study of Atherosclerosis Lung/SNP Health Association Resource Study. *Am. J. Respir. Crit. Care Med.* **189,** 408–418 (2014).

98. Mueller, P. W. *et al.* Genetics of Kidneys in Diabetes (GoKinD) study: a genetics collection available for identifying genetic susceptibility factors for diabetic nephropathy in type 1 diabetes. *J. Am. Soc. Nephrol.* **17,** 1782–1790 (2006).

99. Ober, C. *et al.* Genome-wide association study of plasma lipoprotein(a) levels identifies multiple genes on chromosome 6q. *J. Lipid Res.* **50,** 798–806 (2009).

100. Okada, Y. *et al.* Genetics of rheumatoid arthritis contributes to biology and drug discovery. *Nature* **506,** 376–381 (2014).

101. Olfson, E. & Bierut, L. J. Convergence of genome-wide association and candidate gene studies for alcoholism. *Alcohol. Clin. Exp. Res.* **36,** 2086–2094 (2012).

102. Pankratz, N. *et al.* Copy number variation in familial Parkinson disease. *PLoS One* **6,** e20988 (2011).

103. Amundadottir, L. *et al.* Genome-wide association study identifies variants in the ABO locus associated with susceptibility to pancreatic cancer. *Nat. Genet.* **41,** 986–990 (2009).

104. Schizophrenia Working Group of the Psychiatric Genomics Consortium. Biological insights from 108 schizophrenia-associated genetic loci. *Nature* **511,** 421–427 (2014).

105. Neale, B. M. *et al.* Meta-analysis of genome-wide association studies of attention-deficit/hyperactivity disorder. *J. Am. Acad. Child Adolesc. Psychiatry* **49,** 884–897 (2010).

106. Psychiatric GWAS Consortium Bipolar Disorder Working Group. Large-scale genome-wide association analysis of bipolar disorder identifies a new susceptibility locus near ODZ4. *Nat. Genet.* **43,** 977–983 (2011).

107. Smoller, J. W. *et al.* Identification of risk loci with shared effects on five major psychiatric disorders: a genome-wide analysis. *Lancet* **381,** 1371–1379 (2013).

108. Major Depressive Disorder Working Group of the Psychiatric GWAS Consortium *et al.* A mega-analysis of genome-wide association studies for major depressive disorder. *Mol. Psychiatry* **18,** 497–511 (2013).

109. van Rheenen, W. *et al.* Genome-wide association analyses identify new risk variants and the genetic architecture of amyotrophic lateral sclerosis. *Nat. Genet.* **48,** 1043–1048 (2016).

110. Perry, J. R. *et al.* Parent-of-origin-specific allelic associations among 106 genomic loci for age at menarche. *Nature* **514,** 92–97 (2014).

111. Day, F. R. *et al.* Large-scale genomic analyses link reproductive aging to hypothalamic signaling, breast cancer susceptibility and BRCA1-mediated DNA repair. *Nat. Genet.* **47,** 1294–1303 (2015).

112. Roederer, M. *et al.* The genetic architecture of the human immune system: a bioresource for autoimmunity and disease pathogenesis. *Cell* **161,** 387–403 (2015).

113. Shin, S.-Y. *et al.* An atlas of genetic influences on human blood metabolites. *Nat. Genet.* **46,** 543–550 (2014).

114. Simón-Sánchez, J. *et al.* Genome-wide association study reveals genetic risk underlying Parkinson’s disease. *Nat. Genet.* **41,** 1308–1312 (2009).

115. Smith, E. N. *et al.* Genome-wide association study of bipolar disorder in European American and African American individuals. *Mol. Psychiatry* **14,** 755–763 (2009).

116. Benyamin, B. *et al.* Childhood intelligence is heritable, highly polygenic and associated with FNBP1L. *Mol. Psychiatry* **19,** 253–258 (2014).

117. Rietveld, C. A. *et al.* Common genetic variants associated with cognitive performance identified using the proxy-phenotype method. *Proc. Natl. Acad. Sci. U. S. A.* **111,** 13790–13794 (2014).

118. Rietveld, C. A. *et al.* GWAS of 126,559 Individuals Identifies Genetic Variants Associated with Educational Attainment. *Science* (2013). doi:10.1126/science.1235488

119. Okbay, A. *et al.* Genetic variants associated with subjective well-being, depressive symptoms, and neuroticism identified through genome-wide analyses. *Nat. Genet.* **48,** 624–633 (2016).

120. Barban, N. *et al.* Genome-wide analysis identifies 12 loci influencing human reproductive behavior. *Nat. Genet.* **48,** 1462–1472 (2016).

121. Stahl, E. A. *et al.* Genome-wide association study meta-analysis identifies seven new rheumatoid arthritis risk loci. *Nat. Genet.* **42,** 508–514 (2010).

122. Tobacco and Genetics Consortium. Genome-wide meta-analyses identify multiple loci associated with smoking behavior. *Nat. Genet.* **42,** 441–447 (2010).

123. Tang, C. S. *et al.* Fine mapping of the 9q31 Hirschsprung’s disease locus. *Hum. Genet.* **127,** 675–683 (2010).

124. Trynka, G. *et al.* Dense genotyping identifies and localizes multiple common and rare variant association signals in celiac disease. *Nat. Genet.* **43,** 1193–1201 (2011).

125. Wood, A. R. *et al.* Variants in the FTO and CDKAL1 loci have recessive effects on risk of obesity and type 2 diabetes, respectively. *Diabetologia* **59,** 1214–1221 (2016).

126. Jones, S. E. *et al.* Genome-Wide Association Analyses in 128,266 Individuals Identifies New Morningness and Sleep Duration Loci. *PLoS Genet.* **12,** e1006125 (2016).

127. Pilling, L. C. *et al.* Human longevity is influenced by many genetic variants: evidence from 75,000 UK Biobank participants. *Aging*  **8,** 547–560 (2016).

128. Churchhouse, C. & Neale, B. Rapid GWAS of thousands of phenotypes for 337,000 samples in the UK Biobank. *Neale Lab* (2017). Available at: http://www.nealelab.is/blog/2017/7/19/rapid-gwas-of-thousands-of-phenotypes-for-337000-samples-in-the-uk-biobank. (Accessed: 14th December 2017)

129. Wade, T. D. *et al.* Genetic variants associated with disordered eating. *Int. J. Eat. Disord.* **46,** 594–608 (2013).

130. Toby Johnson, G. S. U. Efficient Calculation for Multi-SNP Genetic Risk Scores. *citeseerx.ist.psu.edu/viewdoc/summary?doi=10.1.1.398.7674*

131. Burgess, S., Butterworth, A. & Thompson, S. G. Mendelian randomization analysis with multiple genetic variants using summarized data. *Genet. Epidemiol.* **37,** 658–665 (2013).

132. Burgess, S., Dudbridge, F. & Thompson, S. G. Combining information on multiple instrumental variables in Mendelian randomization: comparison of allele score and summarized data methods. *Stat. Med.* **35,** 1880–1906 (2016).

133. Pierce, B. L. & Burgess, S. Efficient design for Mendelian randomization studies: subsample and 2-sample instrumental variable estimators. *Am. J. Epidemiol.* **178,** 1177–1184 (2013).

134. Bowden, J., Davey Smith, G., Haycock, P. C. & Burgess, S. Consistent Estimation in Mendelian Randomization with Some Invalid Instruments Using a Weighted Median Estimator. *Genet. Epidemiol.* **40,** 304–314 (2016).

135. Thomas, D. C., Lawlor, D. A. & Thompson, J. R. Re: Estimation of bias in nongenetic observational studies using ‘Mendelian triangulation’ by Bautista et al. *Ann. Epidemiol.* **17,** 511–513 (2007).

136. Hartwig, F. P., Smith, G. D. & Bowden, J. Robust inference in summary data Mendelian randomisation via the zero modal pleiotropy assumption. *bioRxiv* 126102 (2017). doi:10.1101/126102

137. Burgess, S., Freitag, D. F., Khan, H., Gorman, D. N. & Thompson, S. G. Using multivariable Mendelian randomization to disentangle the causal effects of lipid fractions. *PLoS One* **9,** e108891 (2014).

138. Burgess, S., Dudbridge, F. & Thompson, S. G. Re: ‘Multivariable Mendelian Randomization: The Use of Pleiotropic Genetic Variants to Estimate Causal Effects’. *Am. J. Epidemiol.* **181,** 290–291 (2015).

139. Bowden, J., Davey Smith, G. & Burgess, S. Mendelian randomization with invalid instruments: effect estimation and bias detection through Egger regression. *Int. J. Epidemiol.* (2015).

140. Bowden, J. *et al.* A framework for the investigation of pleiotropy in two-sample summary data Mendelian randomization. *Stat. Med.* **36,** 1783–1802 (2017).

141. Lawlor, D. A., Harbord, R. M., Sterne, J. A. C., Timpson, N. & Davey Smith, G. Mendelian randomization: using genes as instruments for making causal inferences in epidemiology. *Stat. Med.* **27,** 1133–1163 (2008).

142. Yavorska, O. O. & Burgess, S. MendelianRandomization: an R package for performing Mendelian randomization analyses using summarized data. *Int. J. Epidemiol.* (2017). doi:10.1093/ije/dyx034

143. Verbanck, M., Chen, C.-Y., Neale, B. & Do, R. Detection of widespread horizontal pleiotropy in causal relationships inferred from Mendelian randomization between complex traits and diseases. *Nat. Genet.* (2018). doi:10.1038/s41588-018-0099-7

144. Zhao, Q., Wang, J., Hemani, G., Bowden, J. & Small, D. S. Statistical inference in two-sample summary-data Mendelian randomization using robust adjusted profile score. *arXiv [stat.AP]* (2018).

145. Greco M, F. D., Minelli, C., Sheehan, N. A. & Thompson, J. R. Detecting pleiotropy in Mendelian randomisation studies with summary data and a continuous outcome. *Stat. Med.* **34,** 2926–2940 (2015).

146. Sterne, J. A. C. *et al.* Recommendations for examining and interpreting funnel plot asymmetry in meta-analyses of randomised controlled trials. *BMJ* **343,** d4002 (2011).

147. Bowden, J. *et al.* Improving the visualisation, interpretation and analysis of two-sample summary data Mendelian randomization via the radial plot and radial regression. *bioRxiv* 200378 (2017). doi:10.1101/200378

148. Nikpay, M. *et al.* A comprehensive 1,000 Genomes-based genome-wide association meta-analysis of coronary artery disease. *Nat. Genet.* **47,** 1121–1130 (2015).

149. Willer, C. J. *et al.* Discovery and refinement of loci associated with lipid levels. *Nat. Genet.* **45,** 1274–1283 (2013).

150. Locke, A. E. *et al.* Genetic studies of body mass index yield new insights for obesity biology. *Nature* **518,** 197–206 (2015).

151. Shungin, D. *et al.* New genetic loci link adipose and insulin biology to body fat distribution. *Nature* **518,** 187–196 (2015).

152. Wain, L. V. *et al.* Genome-wide association study identifies six new loci influencing pulse pressure and mean arterial pressure. *Nat. Genet.* **43,** 1005–1011 (2011).

153. van der Harst, P. *et al.* Seventy-five genetic loci influencing the human red blood cell. *Nature* **492,** 369–375 (2012).

154. Ganesh, S. K. *et al.* Multiple loci influence erythrocyte phenotypes in the CHARGE Consortium. *Nat. Genet.* **41,** 1191–1198 (2009).

155. Gieger, C. *et al.* New gene functions in megakaryopoiesis and platelet formation. *Nature* **480,** 201–208 (2011).

156. Shin, S.-Y. *et al.* An atlas of genetic influences on human blood metabolites. *Nat. Genet.* **46,** 543–550 (2014).

157. Köttgen, A. *et al.* Genome-wide association analyses identify 18 new loci associated with serum urate concentrations. *Nat. Genet.* **45,** 145–154 (2013).

158. Liu, J. Z. *et al.* Association analyses identify 38 susceptibility loci for inflammatory bowel disease and highlight shared genetic risk across populations. *Nat. Genet.* **47,** 979–986 (2015).

159. Okada, Y. *et al.* Genetics of rheumatoid arthritis contributes to biology and drug discovery. *Nature* **506,** 376–381 (2014).

160. Cordell, H. J. *et al.* International genome-wide meta-analysis identifies new primary biliary cirrhosis risk loci and targetable pathogenic pathways. *Nat. Commun.* **6,** 8019 (2015).

161. Dubois, P. C. a. *et al.* Multiple common variants for celiac disease influencing immune gene expression. *Nat. Genet.* **42,** 295–302 (2010).

162. Cordell, H. J. *et al.* Genome-wide association study identifies loci on 12q24 and 13q32 associated with tetralogy of Fallot. *Hum. Mol. Genet.* **22,** 1473–1481 (2013).

163. Zheng, J. *et al.* Recent Developments in Mendelian Randomization Studies. *Curr Epidemiol Rep* **4,** 330–345 (2017).

164. Haycock, P. C. *et al.* Best (but oft-forgotten) practices: the design, analysis, and interpretation of Mendelian randomization studies. *Am. J. Clin. Nutr.* **103,** 965–978 (2016).

165. Davey Smith, G. & Hemani, G. Mendelian randomization: genetic anchors for causal inference in epidemiological studies. *Hum. Mol. Genet.* **23,** R89–98 (2014).

166. Holmes, M. V., Ala-Korpela, M. & Smith, G. D. Mendelian randomization in cardiometabolic disease: challenges in evaluating causality. *Nat. Rev. Cardiol.* **14,** 577–590 (2017).

167. Swerdlow, D. I. *et al.* Selecting instruments for Mendelian randomization in the wake of genome-wide association studies. *Int. J. Epidemiol.* **45,** 1600–1616 (2016).

168. VanderWeele, T. J., Tchetgen Tchetgen, E. J., Cornelis, M. & Kraft, P. Methodological challenges in mendelian randomization. *Epidemiology* **25,** 427–435 (2014).

169. Paternoster, L., Tilling, K. & Davey Smith, G. Genetic epidemiology and Mendelian randomization for informing disease therapeutics: Conceptual and methodological challenges. *PLoS Genet.* **13,** e1006944 (2017).

170. Gage, S. H. *et al.* Assessing causality in associations between cannabis use and schizophrenia risk: a two-sample Mendelian randomization study. *Psychol. Med.* **47,** 971–980 (2017).

171. Power, R. A. *et al.* Genetic predisposition to schizophrenia associated with increased use of cannabis. *Mol. Psychiatry* **19,** 1201–1204 (2014).

172. Gage, S. H., Davey Smith, G. & Munafò, M. R. Schizophrenia and neighbourhood deprivation. *Transl. Psychiatry* **6,** e979 (2016).

173. Munafò, M. R., Tilling, K., Taylor, A. E., Evans, D. M. & Davey Smith, G. Collider scope: when selection bias can substantially influence observed associations. *Int. J. Epidemiol.* (2017). doi:10.1093/ije/dyx206

174. Rohrer, J. M. Thinking Clearly About Correlations and Causation: Graphical Causal Models for Observational Data. *Advances in Methods and Practices in Psychological Science* (2018). doi:10.1177/2515245917745629

175. Lawlor, D. A., Tilling, K. & Davey Smith, G. Triangulation and aetiological epidemiology. *Int. J. Epidemiol.* **in press,** (2016).

176. Saleheen, D. *et al.* Apolipoprotein(a) isoform size, lipoprotein(a) concentration, and coronary artery disease: a mendelian randomisation analysis. *Lancet Diabetes Endocrinol* (2017). doi:10.1016/S2213-8587(17)30088-8

177. Lusis, A. J. & Pajukanta, P. A treasure trove for lipoprotein biology. *Nat. Genet.* **40,** 129–130 (2008).

178. Taylor, A. E. *et al.* Mendelian randomization in health research: using appropriate genetic variants and avoiding biased estimates. *Econ. Hum. Biol.* **13,** 99–106 (2014).

179. Munafò, M. R. *et al.* Association between genetic variants on chromosome 15q25 locus and objective measures of tobacco exposure. *J. Natl. Cancer Inst.* **104,** 740–748 (2012).

180. Aalen, O. O., Valberg, M., Grotmol, T. & Tretli, S. Understanding variation in disease risk: the elusive concept of frailty. *Int. J. Epidemiol.* **44,** 1408–1421 (2015).

181. Noyce, A. J. *et al.* Estimating the causal influence of body mass index on risk of Parkinson disease: A Mendelian randomisation study. *PLoS Med.* **14,** e1002314 (2017).

182. Anderson, E. *et al.* The Causal Effect Of Educational Attainment On Alzheimer’s Disease: A Two-Sample Mendelian Randomization Study. *bioRxiv* 127993 (2017). doi:10.1101/127993

183. Juul, K. *et al.* Genetically reduced antioxidative protection and increased ischemic heart disease risk: The Copenhagen City Heart Study. *Circulation* **109,** 59–65 (2004).

184. Interleukin-6 Receptor Mendelian Randomisation Analysis (IL6R MR) Consortium. The interleukin-6 receptor as a target for prevention of coronary heart disease: a mendelian randomisation analysis. *Lancet* **379,** 1214–1224 (2012).

185. Sarwar, N. *et al.* Interleukin-6 receptor pathways in coronary heart disease: a collaborative meta-analysis of 82 studies. *Lancet* **379,** 1205–1213 (2012).

186. The GTEx Consortium. The Genotype-Tissue Expression (GTEx) pilot analysis: Multitissue gene regulation in humans. *Science* **348,** 648–660 (2015).

187. Bowden, J. *et al.* Assessing the suitability of summary data for two-sample Mendelian randomization analyses using MR-Egger regression: the role of the I2 statistic. *Int. J. Epidemiol.* (2016). doi:10.1093/ije/dyw220

188. Burgess, S., Davies, N. M. & Thompson, S. G. Instrumental variable analysis with a nonlinear exposure-outcome relationship. *Epidemiology* **25,** 877–885 (2014).

189. Staley, J. R. & Burgess, S. Semiparametric methods for estimation of a nonlinear exposure-outcome relationship using instrumental variables with application to Mendelian randomization. *Genet. Epidemiol.* **41,** 341–352 (2017).

190. Burgess, S. Sample size and power calculations in Mendelian randomization with a single instrumental variable and a binary outcome. *Int. J. Epidemiol.* **43,** 922–929 (2014).

191. Hemani, G. *et al.* Automating Mendelian randomization through machine learning to construct a putative causal map of the human phenome. *bioRxiv* 173682 (2017). doi:10.1101/173682

192. Richmond, R. C. *et al.* Assessing Causality in the Association between Child Adiposity and Physical Activity Levels: A Mendelian Randomization Analysis. *PLoS Med.* **11,** e1001618 (2014).

193. Hemani, G., Tilling, K. & Smith, G. D. Orienting The Causal Relationship Between Imprecisely Measured Traits Using Genetic Instruments. *bioRxiv* 117101 (2017). doi:10.1101/117101

194. Bowden, J. & Dudbridge, F. Unbiased estimation of odds ratios: combining genomewide association scans with replication studies. *Genet. Epidemiol.* **33,** 406–418 (2009).
